# Example-Based Super-Resolution Fluorescence Microscopy

**DOI:** 10.1038/s41598-018-24033-7

**Published:** 2018-04-23

**Authors:** Shu Jia, Boran Han, J. Nathan Kutz

**Affiliations:** 1 Department of Biomedical Engineering, Stony Brook University, State University of New York, Stony Brook, New York, USA; 2 Department of Chemistry and Chemical Biology, Harvard University, Cambridge, Massachusetts USA; 3 Department of Applied Mathematics, University of Washington, Seattle, Washington USA

## Abstract

Capturing biological dynamics with high spatiotemporal resolution demands the advancement in imaging technologies. Super-resolution fluorescence microscopy offers spatial resolution surpassing the diffraction limit to resolve near-molecular-level details. While various strategies have been reported to improve the temporal resolution of super-resolution imaging, all super-resolution techniques are still fundamentally limited by the trade-off associated with the longer image acquisition time that is needed to achieve higher spatial information. Here, we demonstrated an example-based, computational method that aims to obtain super-resolution images using conventional imaging without increasing the imaging time. With a low-resolution image input, the method provides an estimate of its super-resolution image based on an example database that contains super- and low-resolution image pairs of biological structures of interest. The computational imaging of cellular microtubules agrees approximately with the experimental super-resolution STORM results. This new approach may offer potential improvements in temporal resolution for experimental super-resolution fluorescence microscopy and provide a new path for large-data aided biomedical imaging.

## Introduction

Super-resolution fluorescence imaging techniques have overcome the optical diffraction limit of conventional fluorescence microscopy, allowing visualization of biological structures with near-molecular-scale resolution^1,2^. However, an inevitable challenge for all super-resolution techniques remains that greater spatial resolution is obtained at the expense of prolonged acquisition time, leading to compromised temporal resolution for live imaging. For wide-field techniques based on single-molecule localization, such as stochastic optical reconstruction microscopy (STORM)^3^ or (fluorescence) photoactivated localization microscopy ((F)PALM)^4,5^, this trade-off arises from the need to accumulate enough single-molecule localizations to reveal the biological structures (i.e. to meet Nyquist criterion, in which the image sampling interval must be smaller than half of the desired resolution)^6^. For methods based on patterned-illumination, such as stimulated emission depletion (STED) microscopy^7^ and (saturated) structured illumination microscopy ((S)SIM)^8^, this compromise is caused by longer scanning time or the need to acquire images with a series of excitation patterns. Various strategies have been reported to accelerate these super-resolution imaging processes without significantly degrading the spatial resolution. For STORM or (F)PALM, the methods include those that increase the single-molecule switching rate by stronger excitation and the readout speed with faster cameras^9,10^ and those that allow a higher density of emitted fluorophores in each individual frame by discerning the overlapping single-molecule images with computational algorithms, such as DAOSTORM^11,12^, Bayesian statistics^13^ and compressed sensing^14,15^. For STED or SIM, the improvement in temporal resolution has been focused on the parallelization of multiple patterns in one camera exposure in order to reduce the total imaging time^16,17^. While these approaches have led to substantial improvements in temporal resolution, by orders of magnitude in some cases, the construction of a sub-diffraction-limit image is still fundamentally limited by the need to accumulate spatial information in a sequential fashion. Here we present an example-based approach to inferring a super-resolution (SR) image directly from a single low-resolution (LR) fluorescent image for those structures with prior knowledge of their shapes. With a sufficiently large example library that contains images of structures with a large variety of different shapes, it might also be possible to use this method to infer SR images for structures without prior knowledge of their shapes. This method has a potential to advance the way by which images are obtained in super-resolution microscopy, thereby significantly improving temporal resolution.

Example-based resolution improvement methods have recently emerged in computer vision, whereby a LR natural image (e.g. a photograph) can be efficiently transformed into high-resolution format by learning and estimating from a database that consists of low- and high-resolution example images^18^. These example images of natural scenes are typically unrelated to the LR image of interest, and the resolution improvement relies on the statistical restoration of the missing high-frequency components in the LR image by inference from the features of the frequency compositions in the examples^18^. In contrast to natural images, fluorescent images are highly specified, exhibiting precisely labeled molecular targets or structures in the sample. Thus, for fluorescence microscopy, the LR and SR examples in the database can be composed of fluorescent images consisting of the same type of biological structures as in the LR image of interest, so that more precise (less statistical) image inference can be achieved.

**Figure 1:**
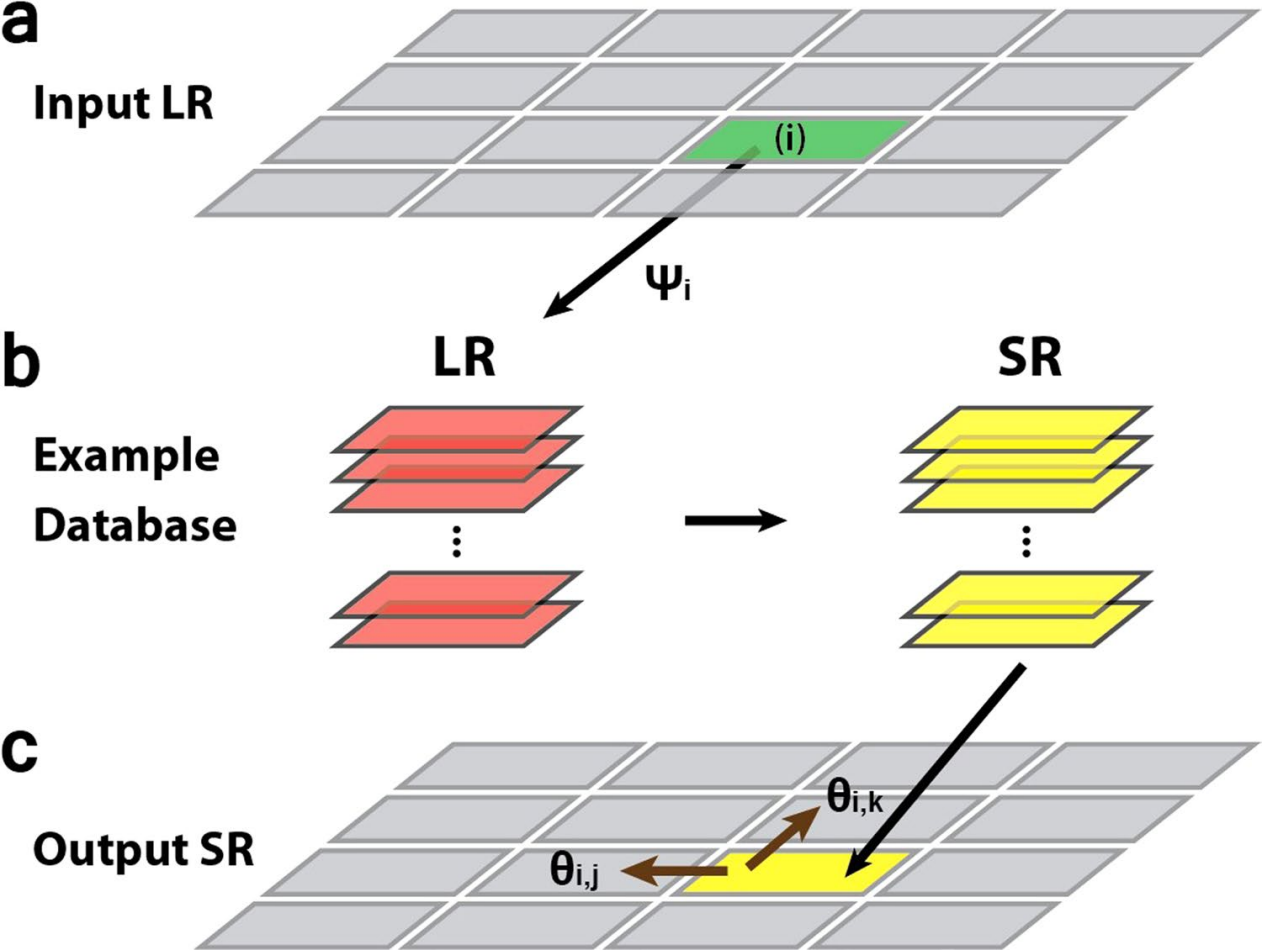
Workflow of the example-based resolution improvement method. (**a**) An input low-resolution (LR) image is segmented into patches. (**b**) For a representative patch (i) of the input LR image, its LR matches in the database are selected based on the evidence potential *ψ*_*i*_(*x*_*i*_) (see Methods), along with their corresponding SR image counterparts. (**c**) The selection of the best SR candidates considers the overlapping potential *θ*_*ij*_ and *θ*_*ik*_ between neighboring SR candidate patches. These selected SR patches are pieced together to reconstruct the final output SR image.

The principle of our method is shown in Fig. 1 (for details, see Methods and Supplementary Figure 1). First, LR and SR example image pairs are segmented into small patches and stored in a database. Next, an input LR image is also segmented into patches with the same size as those in the example database (Fig. 1a). Third, to infer the SR form of the LR input, we first compared each individual patch of the input LR image with the LR patches in the example database. The pixel-value distances between these patches are calculated, and those LR example patches that have the lowest distance (i.e. highest similarity), along with their SR pairs, are selected as candidates (Fig. 1b). Finally, the pixel-value distance between the overlapping boundaries of the neighboring selected SR candidates are calculated. Those that provide the overall smoothest connectivity are chosen to construct the final SR image (Fig. 1c). These two steps of inference are described by a Markov random field (MRF). A detailed mathematical description of the procedures can be found in Methods.

**Figure 2:**
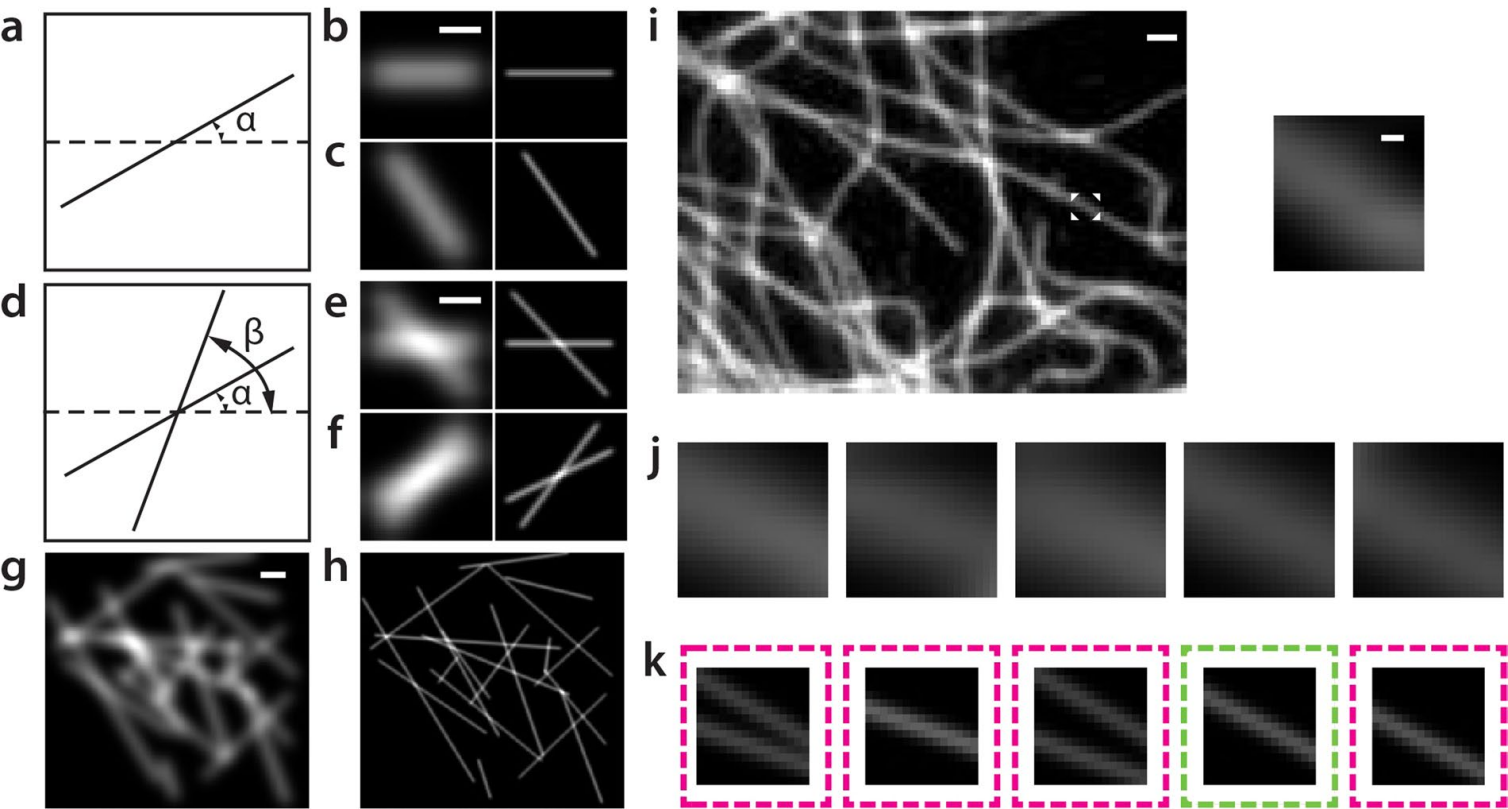
Construction of the example database of microtubule filaments. (**a**–**h**) Example database of synthetic microtubules. The database is composed of three categories: (**a**–**c**) single microtubules oriented at various angles α (rotating from 0° to 178° at steps of 2°); (**d**–**f**) two crossing microtubules oriented at various angles α and β (individually rotating from 0° to 178° at steps of 2°, α ≠ β); (**g**,**h**) representative randomly distributed microtubules. For an input LR experimental image shown in (**i**), one of its segmented patches (boxed region in (**i**), magnified in the inset) is matched with several LR candidates in the database (**j**). High similarity is observed between these LR candidates and the input patch (the inset in (**i**)). The corresponding SR candidates (**k**), however, display distinct differences. The dashed boxes in (**k**) outline the edges of LR candidates. The SR image boxed in the green dashed box represents the selected candidate that best fulfills the overall connectivity in the final SR image of (**i**). Scale bars, (**b**,**e**,**g**) 500 nm; (**i**) 1 µm; the inset of (**i**) 100 nm.

**Figure 3:**
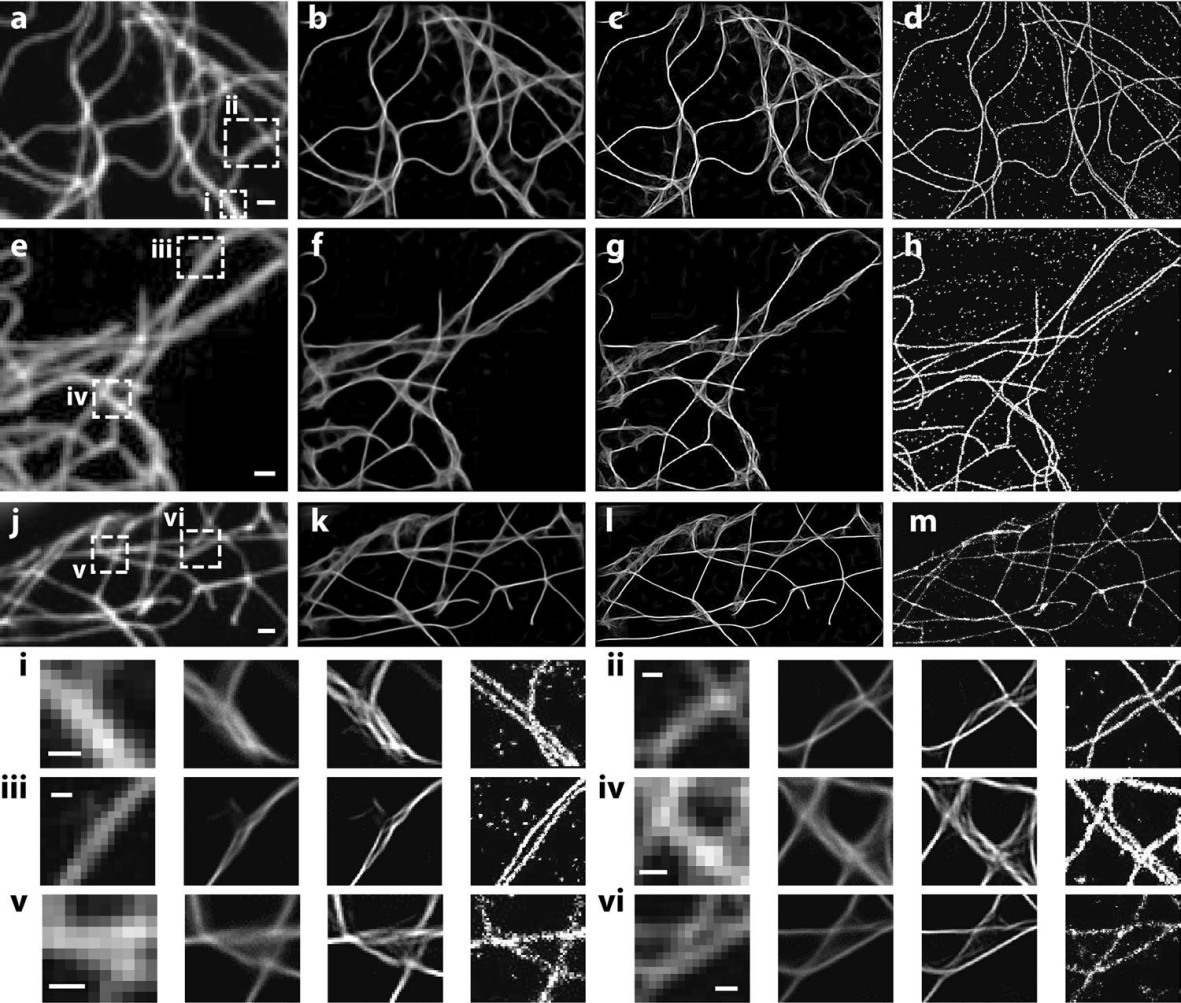
Super-resolution reconstruction of microtubule filaments. (**a**,**e**,**j**) Experimental LR input images of microtubules. (**b**,**f**,**k**) The respective computational SR images using our example-based method. (**c**,**g**,**l**) The contrast-enhanced images of (**b,f**,**k**), respectively. (**d,h,m**) Corresponding STORM images of the same areas, respectively. (i–vi) The corresponding magnified boxed regions in (**a,e,j**), respectively. Scale bars, (**a,e,j**) 1 µm, (i–vi) 500 nm.

Experimentally, we validated the method by imaging microtubules in cells, a biological structure often used for the calibration of SR imaging systems. To build the example database, we considered the known width and morphology of fluorescently labeled microtubules^19^ and developed a strategy to construct a synthetic database (see Methods). We reason that the use of synthetic images, rather than experimental ones, can substantially reduce redundancy in the database and provide the capability to address any specific (or complex) structures that require corresponding examples in the database. Practically, we categorized the LR and SR example image pairs of microtubules into three groups based on their geometrical features: 1) single microtubule filaments of varying orientations (Fig. 2a–c), 2) two crossing microtubule filaments of varying orientations (Fig. 2d–f), and 3) randomly distributed microtubule filaments (Fig. 2g,h). The first two groups covered a large portion of microtubule features due to the sparse and extended microtubule distributions in cells. The third group is created to remedy those missing features from the first two groups, e.g. to consider the cases of densely packed regions with more than two filaments. In this work, we limited the number of images in the third group for demonstration. As seen later, however, we showed that the performance of the method can be substantially improved by expanding the third group to contain more microtubule features.

Next, once the database of microtubule filament images is established, a LR image of interest was introduced as shown in Fig. 2i, and the two-step procedures of image inference described above were applied. As a specific example, first, the LR input image was segmented into patches, which were compared with the database to search for matching LR and SR candidates. The search returned the best 30 LR candidates as well as their SR pairs. As shown in Fig. 2j, these LR candidates were largely indistinguishable due to the limited resolution, while their SR images were explicitly distinct from each other (Fig. 2k). Thus, the second step of inference was performed to choose among these SR candidates based on the connectivity between their boundaries. The 30 SR candidates were examined by comparing the overlapping regions of neighboring patches in both horizontal and vertical dimensions (as previously shown in Fig. 1c). Among various combinations formed by these SR candidates, the one that fulfilled the best global (i.e. whole image) connectivity was finally selected to construct the SR output image (see Methods). It should also be noted that the example database as shown in Fig. 2a–h only considered rotational rather than translational variance among microtubule features. For example, in Fig. 2a–c, the translations of the single microtubule filaments oriented in varying angles in each example image were not considered (i.e. these filaments are all centered in each example image). This may result in less accurate inference to cover all possible microtubule distributions. To address this problem without increasing the size of the database, i.e. the computation time, we combined another strategy. Here we instead translated the input LR image at single-pixel steps, performed the two-step image inference to identify the SR images of all the translated images, and then translated the SR results inversely by the same amount of single-pixel steps (Supplementary Figure 2). The final SR image of the original LR input is the average over all these SR images. Since the image inference procedures maximally search for the best SR reconstruction from the database, this average provides the maximum likelihood of the accurate reconstruction of all patches. As a result, this strategy effectively enhances the usefulness of a relatively small database, overcomes the lack of sufficient examples of translations, and provides both smoothness and accuracy in the final SR image (Supplementary Figure 3).

Figure 3 shows several super-resolved microtubule images using this resolution improvement method. Compared with images taken by the conventional LR wide-field microscope (left column, Fig. 3), the computational results using our method exhibit considerably improved resolution (center columns, Fig. 3). As seen in the magnified sections, some of originally indiscernible microtubule filaments in the conventional images became resolved using the computational method. We also compared the results with experimentally determined super-resolution images of the same areas using STORM (right column, Fig. 3). The global microtubule structures obtained by the example-based, computational method approximately agreed with the experimental super-resolution imaging results. However, it is worth noting that compared with the STORM results, some incorrectly retrieved SR features, especially at those dense structures, can be clearly noticed.

**Figure 4:**
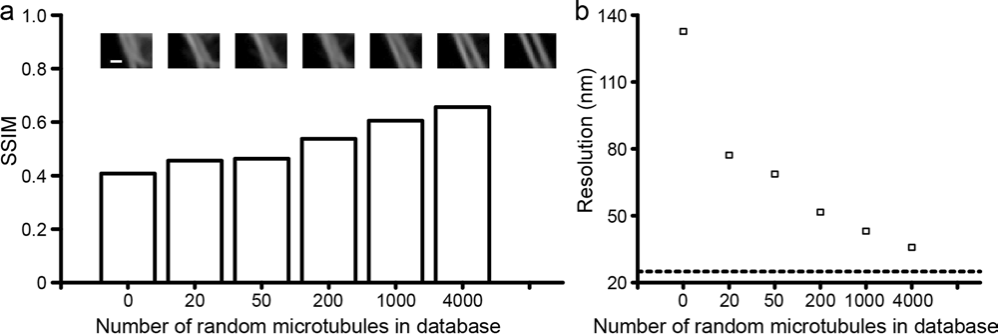
Performance of the method with varying database size. (**a**) Structural similarity index measurement (SSIM) as a function of the number of random microtubules in the simulated database (from 0 to 4000). SSIM = 1 would represent a complete recovery of the original SR object. The values of SSIM were measured over several images. The insets show representative output SR images of two close-by filaments obtained with different database size, respectively. The rightmost inset shows the original SR object. Scale bar, 200 nm. (**b**) Corresponding spatial resolution as a function of database size, measured over several images. The dashed line represents the 25-nm theoretical resolution used for the generation of the SR examples computationally in the database.

We find that the completeness of the database is critical to the performance of the method. The demonstration in Fig. 3 utilized only 30 images in the third group as described in Fig. 2, which led to a substantial lack of necessary features for the reconstruction. One could be the lack of more complex examples that resulted in erroneous inference for high-density structures. Also, the database is lacking three-dimensional (3D) examples, so the algorithm may confuse the image of a defocused filament (i.e. broadened image due to the diffraction of light) with that of two or more overlapping filaments. To examine such a dependence on the completeness of the example database, we show in Fig. 4a that nearby filaments became better resolved as we moderately enlarged the number of randomly distributed microtubules in the database. Quantitatively, we evaluated this improved image inference by the structural similarity index (SSIM)^20^, which is used as a universal image quality measure to compare processed and ground-truth images. Here the computational images obtained by our example-based method were compared with the experimentally derived SR images as a function of the database size (see Methods). The comparison showed that the SSIM increased for databases containing more examples, a quantitative result consistent with the visually apparent improvements in image quality seen with larger databases. In addition, we also measured the closest distance between microtubules that the computational method could resolve correctly. We used this value to determine the resolution in our computational imaging system. As shown in Fig. 4b, the system resolution improves as the database is enriched with more features. We also showed in Supplementary Figure 4 the stability of the inference by calculating the standard deviation (SD) of the matches as we translated the input LR image at the single-pixel step. This SD describes the consistence of the inference around a certain pixel as its containing patch is translated. In the case when the library is built with sufficient true structures that match the structures under investigation, all these patches should be able to find identical matches. Hence, around such a location, the SD will be low and the inference is thus considered to be highly reliable. All these results imply that the performance of the method is critically dependent on the composition of the database. Furthermore, noise is another factor that affects the performance of the method. In practice, the noise level (or signal-to-noise ratio (SNR)) in the library should be consistent with that in the input LR image so that the images can be fairly compared. As shown in Supplementary Figure 5, the quality of reconstruction can be much improved when the SNRs in the library and input are similar, while the quality becomes degraded when the SNRs are deviated. In addition, the higher the SNRs in both the library and input, and better the quality of the computed image. The finding implies the critical role of the noise level and in the future development of the method. In addition to simply increasing the number of features present in the library, we could also strategically enhance the database with tailored features based on the regions in the final SR image where extra computational consideration is required. Such an algorithm could adaptively generate examples in the library focusing on a specific complex structure in the sample. For example, if a LR image has a region that shows the crossing of five microtubules, the algorithm should be able to adaptively learn and generate a large set of examples to infer this case until a satisfying quantitative score is reached.

In summary, our proof-of-principle work demonstrates a resolution improvement method that is able to computationally convert a LR image into its SR form with an algorithm using a database of examples. The work reveals that SR images can be obtained directly from their LR images without the need to accumulate sequential image frames in some cases. We anticipate this approach to work better for those structures that prior knowledge of their shapes exists, which would facilitate the construction of the example library. We thus expect this computation-based method to improve image resolution from conventional LR images to be useful for imaging fast dynamics of known biological structures (e.g. microtubule dynamics) with higher resolution. Furthermore, compared with deconvolution microscopy, because this method explores both prior knowledge and boundary (or global) connectivity, the resulting images are not only sharpened but also able to resolve some indiscernible (overlapping) structures where deconvolution is unable to resolve due to the lack of a prior knowledge based algorithmic framework (Supplementary Figure 6). It is however important to note that, though greater resolution has been achieved with this example-base computational method, inaccurately retrieved patterns have also been observed compared to the experimentally obtained super-resolution images. Practically, labeling deficiencies will affect the performance of the method due to both the non-uniform brightness in the structure and noise in the background. In the current work because we used a simulated library, which brightness is uniform and background contains no noise. Future improvements can be made by additionally include experimental examples in the library which may partially mitigate the problem. In addition, the current implementation of the method may not be able to identify new structures if they do not have corresponding examples in the library, or the global smoothness may not be well achieved for structures that contain spatially separated clusters, rather than continuous ones like microtubules. These reflect limitations in both the database and the algorithm. Future improvements on both fronts may help overcome this challenge and allow the method to be used for broader applications. For example, future development will be aimed at developing a hybrid database that efficiently combines numerical simulations that take into account prior knowledge of the sample of interest with the experimental results obtained by all types of super-resolution optical imaging techniques (STORM/(F)PALM, STED or SIM, etc.). To further broaden the structures that can be inferred, we will establish a collection of databases for many types of biological structures, as well as develop algorithms to address local features (e.g. geometry, polarization, orientation) in addition to global connectivity, so that the method can be adapted to a wide range of biological investigations. With a sufficiently large example library that increase the possibility that the shapes of the structures under investigation are adequately covered in the library, this approach might allow us to approximate SR images of structures from LR conventional images without prior knowledge of their shapes. Computationally, the current method is demonstrated on a desk-top PC (2.67-GHz GPU, 72-GB Memory), and the reconstruction of each SR image requires 5–10 hours. Accelerated computing approaches will be incorporated into the existing algorithm for future development. In the long term, we expect to establish an on-line system where all researchers in the field can contribute their image data to study specific biological structures. More advanced algorithms will be developed to sort and process the on-line data, construct such libraries, and perform SR image reconstruction. With further advancement, this example-based approach may enable computational super-resolution imaging of many biological structures and provide a new path for large-imaging-data aided biomedical research.

## Methods

### The principle of the algorithm

The input LR image of interest is represented by *p*, which contains two-dimensional pixel values of the image. For the first procedure, for patch *i* in the input LR image *p*, the images of its LR matching candidates *x*_*i*_ are denoted by *L*(*x*_*i*_). Based on a Markov random field (MRF), we consider that any of the example patches have an equal probability to match the input patch *i*. Thus, the difference between the input and example patches is considered as independent, equally distributed Gaussian noise at each pixel. The evidence potential *ψ*_*i*_(*x*_*i*_) is then described as

$${{\psi }}_{i}({x}_{i})=\exp (\,-\,|p-L({x}_{i}){|}^{2}/(2{{\sigma }}_{1}^{2})),$$

where the variance *σ*_1_ is determined by the completeness of the example database, e.g. a large example database yields better matching and hence a smaller variance. In this work, we used a normalized *σ*_1_ = 1 for all measurements. Based on the evidence potential, the best matching LR and SR candidates are selected. For the second procedure, belief propagations between the neighboring SR candidate patches are performed to ensure globally smooth connectivity. As shown in the following Appendix section and Supplementary Figure 1 on database construction, each SR patch has an overlapping region with the neighboring ones. Hence, the selection of the best candidate in the second procedure relies on the connectivity potential ${\theta }_{ij}({x}_{i},{x}_{j})$ between neighboring SR candidate patches *S*(*x*_*i*_) and *S*(*x*_*j*_) for input patches *i* and *j*, respectively. Again, we assume the difference of the overlapping region *O*_*ij*_(or *O*_*ji*_) of the SR candidates are described by MRF and hence follows a Gaussian distribution. Thus

$${{\theta }}_{ij}({x}_{i},{x}_{j})=\exp (\,-\,|{O}_{ij}(S({x}_{i}))-{O}_{ji}(S({x}_{j})){|}^{2}/(2{{\sigma }}_{2}^{2}))$$

where *σ*_2_ denotes the variance of difference between the overlapping regions in neighboring candidates, which is set to 1 in this work for all measurements. With these two procedures, the goal is then to globally maximize the joint probability

$$P(x)=\frac{1}{Z}\prod _{i}{{\psi }}_{i}({x}_{i})\prod _{(i.j)\in {\varepsilon }}{{\varphi }}_{ij}({x}_{i},{x}_{j})$$

where *ε* is the set of edges in the above MRF procedures denoted by the neighboring patches *i* and *j*, and Z is a constant normalization factor. Those SR candidate patches that fulfill a maximized joint probability *P*(*x*) will be selected to construct a final SR image.

### The construction of synthetic microtubules

As shown in Fig. 2, numerically simulated LR and SR image pairs of microtubule filaments were generated in three categories: single filaments, crossing filaments, and randomly distributed filaments. The LR and SR microtubules have a 341-nm and 60-nm width, respectively, consistent with experimental measurement of immuno-stained microtubules^19^. The orientations of these microtubules are described in the main text and the caption of Fig. 2. In the third category, each microtubule is determined by its two ends, the positions of which are randomly chosen within the field of the image. Each image contains 20 such randomly distributed microtubules. LR example images are 50 pixel × 50 pixel in categories 1 and 2, and 200 pixel × 200 pixel in category 3, with a pixel size = 160 nm. Corresponding SR example images are 250 pixel × 250 pixel in categories 1 and 2, and 1000 pixel × 1000 pixel in category 3, with a pixel size = 32 nm. In this work, background noise in both LR and SR images were ignored given the fact that the labeling is highly specified and the use of relatively thin samples (compared to the depth of focus) in STORM.

### The construction of database

First, LR example images as described above were up-sampled 5 times using bi-cubic interpolation so that the pixel size in the LR images (160 nm/5 = 32 nm) matched that in their SR counterparts (32 nm). Next, LR images were segmented into 21-pixel × 21-pixel patches, with each segmentation centered at a step of 5 pixels in both horizontal and vertical dimensions (Supplementary Figure 1). As a result, the overlapping regions of neighboring patches are 21 pixel × 16 pixel. The central 15-pixel × 15-pixel region of each LR patch was paired with a SR image. As a result, the adjacent SR patches have an overlapping region of 15 pixel × 10 pixel. Both LR and SR patches were stored as k-dimensional (k-d) binary tree^21^ for data sorting and searching.

### The segmentation of an input LR image

An input image (camera pixel size = 160 nm) was first up-sampled 5 times using bi-cubic interpolation to match the pixel size of the LR and SR patches in the database (32 nm). The image was then segmented into 21-pixel × 21-pixel patches, centered at a step of 12 pixels in both horizontal and vertical dimensions (Supplementary Figure 1). As a result, neighboring patches have an overlapping region of 21 pixel × 9 pixel. After the first procedure, SR candidate patches for each input patch have an overlapping region of 15 pixel × 3 pixel. The determination of the above patch size in both the database and the input image is based on the empirical consideration that it is difficult to find matching examples for a larger patch in the database due to the complicated structural features, or a smaller patch that contains too little information for the algorithm to process accurately. Supplementary Figure 7 shows the outcome of different patch sizes. In addition, because SR images reveal more information than LR ones, the SR patches are thus designed to be smaller than the LR ones (i.e. using the central 15-pixel × 15-pixel region of each LR patch).

### Image contrast enhancement

To provide a better image contrast in Fig. 3, we employed a linear filter provided in MATLAB to extract gradient information (i.e. sharpness) from the images. In brief, the filter adopts a standard 2D Laplacian derivative operator^22^: $$(\begin{array}{ccc}1 & 1 & 1\\ 1 & -8 & 1\\ 1 & 1 & 1\end{array})$$. Under this operator, a 2D image f at pixel (i, j) becomes

$$\begin{array}{c}[f(i+1,j)+f(i-1,j)+f(i,j-1)+f(i,j+1)+f(i+1,j+1)\\ \,+f(i+1,j-1)+f(i-1,j-1)+f(i-1,j+1)-8f(i,j)]\end{array}$$

Such an image contrast enhancement improves the image quality but not super-resolution capability.

### Structural similarity index (SSIM)

The index is made of three comparisons: luminance, contrast and structure^20^. In this work, the index mainly considers structural comparison using correlation between the two images. Other factors such as luminance and contrast are negligible due to the use of synthetic data. We used the standard SSIM function in MATLAB to quantify the quality of SR reconstructions in Fig. 4.

### Immunofluorescence staining of microtubules

STORM sample preparation, imaging and data analysis follow previously reported procedures^19^. In brief, immunostaining was performed using BS-C-1 cells (American Type Culture Collection) cultured with Eagle’s Minimum Essential Medium supplemented with 10% fetal bovine serum, penicillin and streptomycin, and incubated at 37 °C with 5% CO_2_. Cells were plated in LabTek 8-well coverglass chambers at ~20,000 cells per well 18–24 hours prior to fixation. The immunostaining procedure for microtubules and mitochondria consisted of fixation for 10 min with 3% paraformaldehyde and 0.1% glutaraldehyde in PBS, washing with PBS, reduction for 7 min with 0.1% sodium borohydride in PBS to reduce background fluorescence, washing with PBS, blocking and permeabilization for 20 min in PBS containing 3% bovine serum albumin and 0.5% (v/v) Triton X-100 (blocking buffer (BB)), staining for 40 min with primary antibody (rat anti-tubulin (ab6160, Abcam) for tubulin or rabbit anti-TOM20 (sc-11415, Santa Cruz) for mitochondria) diluted in BB to a concentration of 2 μg/mL, washing with PBS containing 0.2% bovine serum albumin and 0.1% (v/v) Triton X-100 (washing buffer, WB), incubation for 30 min with secondary antibodies (~1–2 Alexa 647 dyes per antibody, donkey anti-rat for microtubules and donkey anti-rabbit for mitochondria, using an antibody labeling procedure previously described)^19^ at a concentration of ~2.5 μg/mL in BB, washing with WB and sequentially with PBS, postfixation for 10 min with 3% paraformaldehyde and 0.1% glutaraldehyde in PBS, and finally washing with PBS.

### STORM imaging buffer

All imaging was performed in a solution that contained 100 mM Tris (pH 8.0), an oxygen scavenging system (0.5 mg/mL glucose oxidase (Sigma-Aldrich), 40 μg/mL catalase (Roche or Sigma-Aldrich) and 5% (w/v) glucose) and 143 mM beta-mercaptoethanol.

### STORM imaging

A 647-nm laser at an intensity of 2 kW cm^−2^ was used for excitation of the dyes. Under this condition, the dye molecules were in the fluorescent state initially but rapidly switched to a dark state. All STORM movies were recorded at a frame rate of 60 Hz using home-written Python-based data acquisition software. The movies typically consisted of 30,000–100,000 frames. During each movie, a 405-nm laser light (ramped between 0.1 and 2 W cm^−2^) was used to activate fluorophores and to maintain a roughly constant density of activated molecules. STORM data was analyzed using lab-written software.

## Electronic supplementary material

Supplementary Information
