# Supplementary material for: Example-Based Super-Resolution Fluorescence Microscopy
Source: Sci Rep. 2018 Apr 23;8:5700. doi: 10.1038/s41598-018-24033-7 (PMC5913229; doi:10.1038/s41598-018-24033-7)
Supplement: Supplementary file 1 — Supplementary Information [file 41598_2018_24033_MOESM1_ESM.pdf]

Supplementary Information for

Example-based Super-Resolution Fluorescence Microscopy

Shu Jia, Boran Han, J. Nathan Kutz

Supplementary Figure 1	Illustration of image segmentation in the database and input LR image.
Supplementary Figure 2	LR image translation for better SR reconstruction
Supplementary Figure 3	Comparison between SR results without and with the translation & average approach
Supplementary Figure 4	Quantitative evaluation of reconstruction
Supplementary Figure 5	Performance of the method with varying noise levels
Supplementary Figure 6	Comparison with image deconvolution
Supplementary Figure 7	Performance of the method with varying patch sizes.

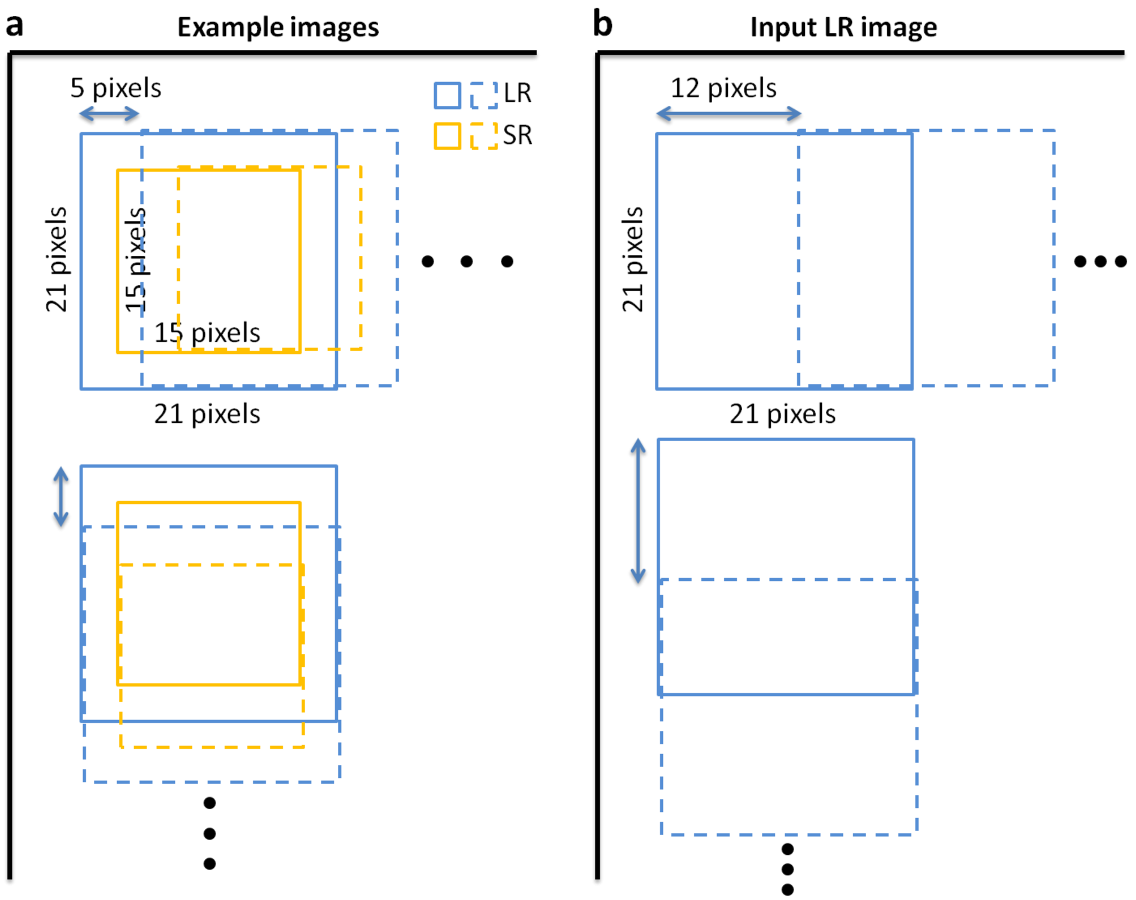

Supplementary Figure 1. Illustration of image segmentation in the database (a) and input LR image (b). (a) LR images in the database were segmented into 21-pixel x 21-pixel patches, centered at a step of 5 pixels in both horizontal and vertical dimensions. The overlapping region of neighboring LR patches is thus 21 pixel x 16 pixel. The central 15-pixel x 15-pixel region of each LR patch was paired with a SR image patch. The adjacent SR patches thus have an overlapping region of 15 pixel x 10 pixel. (b) The input LR image was segmented into 21-pixel x 21-pixel patches, centered at a step of 12 pixels in both horizontal and vertical dimensions. As a result, neighboring patches have a 21-pixel x 9-pixel overlapping region. SR candidate patches for each input patch have a 15-pixel x 3-pixel overlapping region. Solid and dashed boxes represent neighboring patches in both horizontal and vertical dimensions.

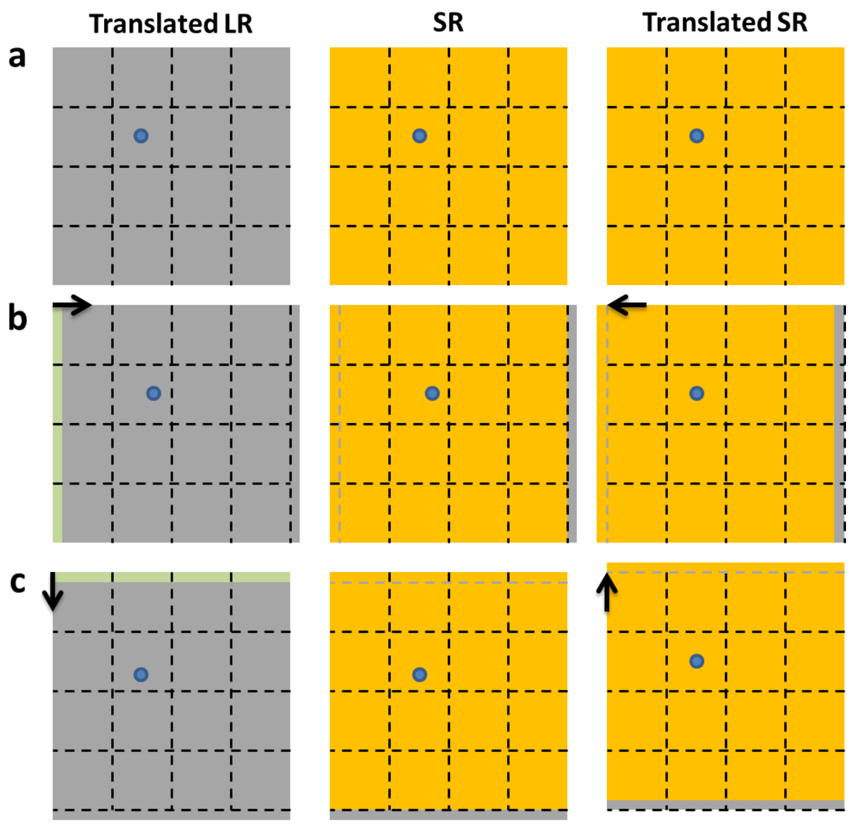

Supplementary Figure 2. LR image translation for better SR reconstruction. Columns from left to right represent translated LR images, SR images based on the translated LR images, and inversely translated SR images, respectively. (a) LR image with translation distance = 0. (b) LR image with a translation in the horizontal direction. (c) LR image with a translation in the vertical direction. Dashed lines represent patch boundaries (simplified to ignore overlapping regions). In the case of (b) or (c), the entire LR image is first translated in the direction indicated by the arrow. The region that is moved out of the original image area will not be considered for reconstruction. A blank region (green bar) is then added to the translated image to compensate the patches on the boundary so that all patches have the same size. These outer patches of the LR image will be removed in the final reconstructed image, which is feasible because the patch size is insignificant compared to the entire image. After being processed by the method, the SR image will be translated back to the original position as indicated by the arrow. The final SR image is constructed by averaging over all the SR images translated at different distances. Maximal translation distance in both horizontal and vertical dimensions is 21 pixels (the size of a LR patch). The blue dots are used to illustrate relevant positional changes. Better SR reconstruction is achieved with this approach, as shown in Supplementary Figure 3.

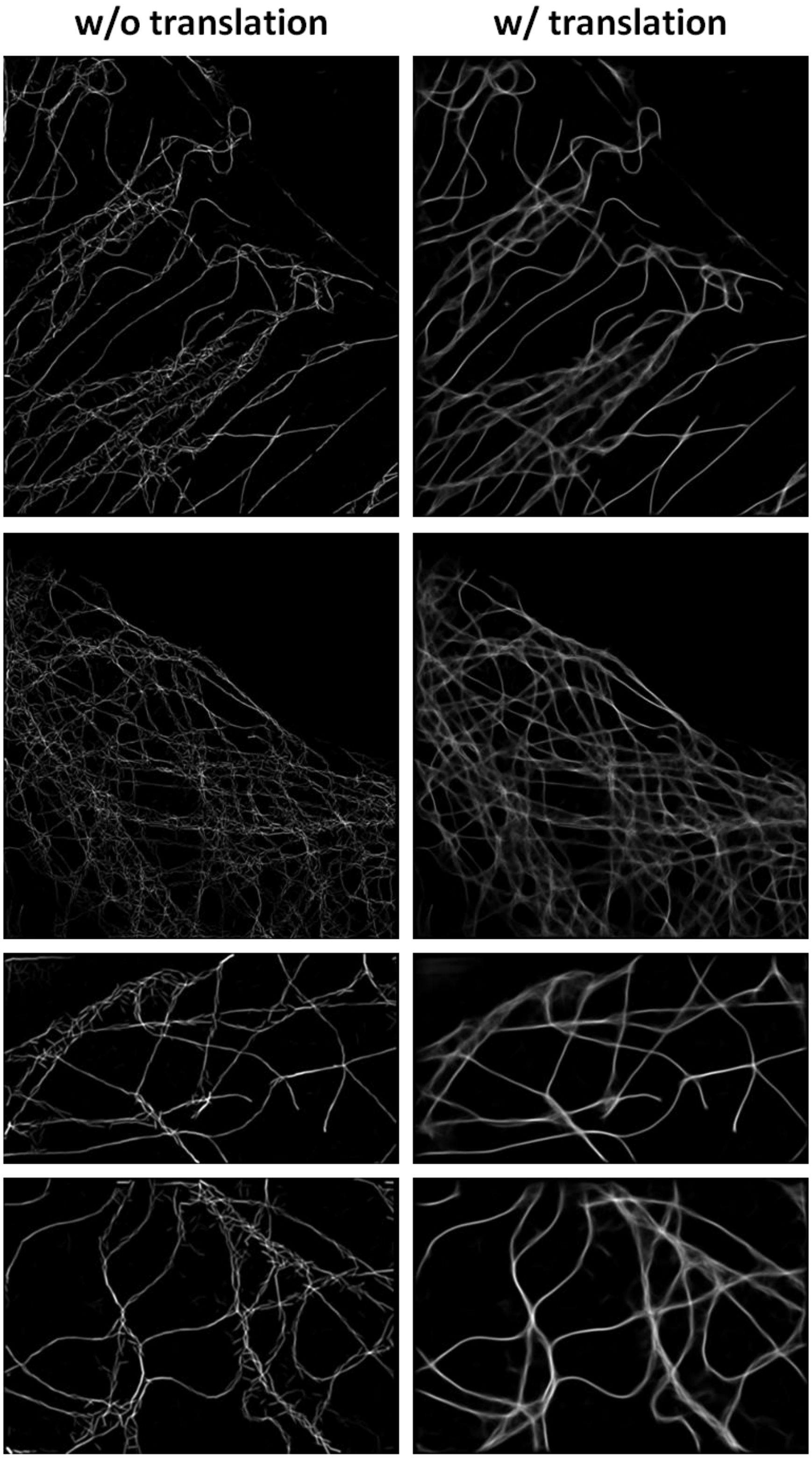

Supplementary Figure 3. Comparison between SR results without and with the translation & average approach. Left column, without translation; right column, with translation and average. As seen, without the process of translation and average, the method leads to inaccurate and discontinuous SR image reconstruction especially at fine structures.

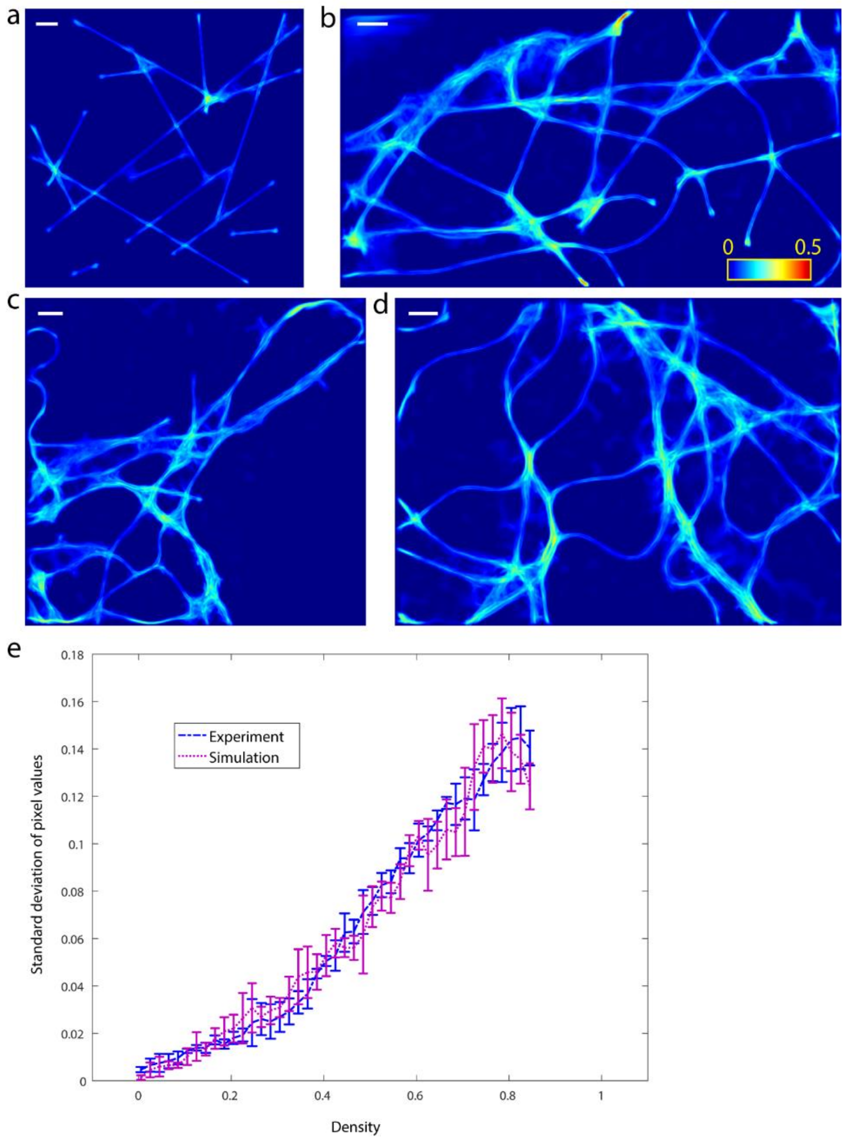

Supplementary Figure 4. Quantitative evaluation of reconstruction. The stability of the inference is quantified by calculating the standard deviation (SD) of the matches as we translated the input LR image at the single-pixel step. This SD describes the consistence of the inference around a certain pixel as its containing patch is translated. In the case when the library is built with sufficient true structures, all these patches should be able to find identical matches. Hence, around such a location, the SD will be low and the inference is thus considered to be highly reliable. (a) The color-map of the SD using simulated data, indicating that the high-density region typically has a relatively high SD. (b-d) The color-map of the SD of Figure 3b-k, respectively. (e) Relationship between the SD and the density of structure at each pixel. The density is defined by the ratio of the area occupied by a valid structure over the entire patch area (e.g. density = 1 represents a structure covers the entire patch). Agreement is shown between the experimental and numerical data. Scale bars: 1μm.

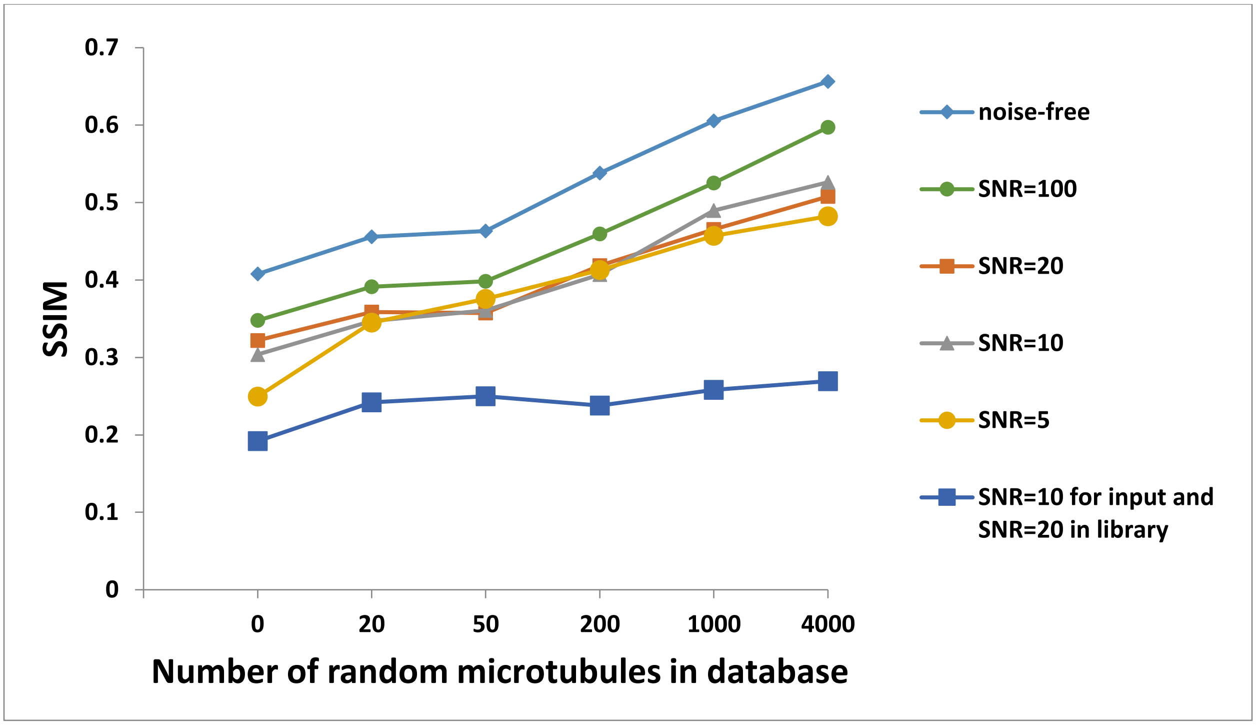

Supplementary Figure 5. Performance of the method with varying noise levels. Structural similarity index measurement (SSIM) as a function of the number of random microtubules in the database (from 0 to 4000) with noise levels in both the database and input image (represented by signal-to-noise-ratio or SNR) at zero (cyan), 5 (yellow), 10 (gray), 20 (orange), and 100 (green), respectively. The blue curve shows the SSIM with different noise levels in the database and input image (20 and 10, respectively). It is shown that when the SNRs are the same in the database and input, as the library size increases, the improvement of the reconstruction follows a similar slope compared to the noise-free case. However, when SNRs deviate in the library and the input, the SSIM becomes worse and barely improves.

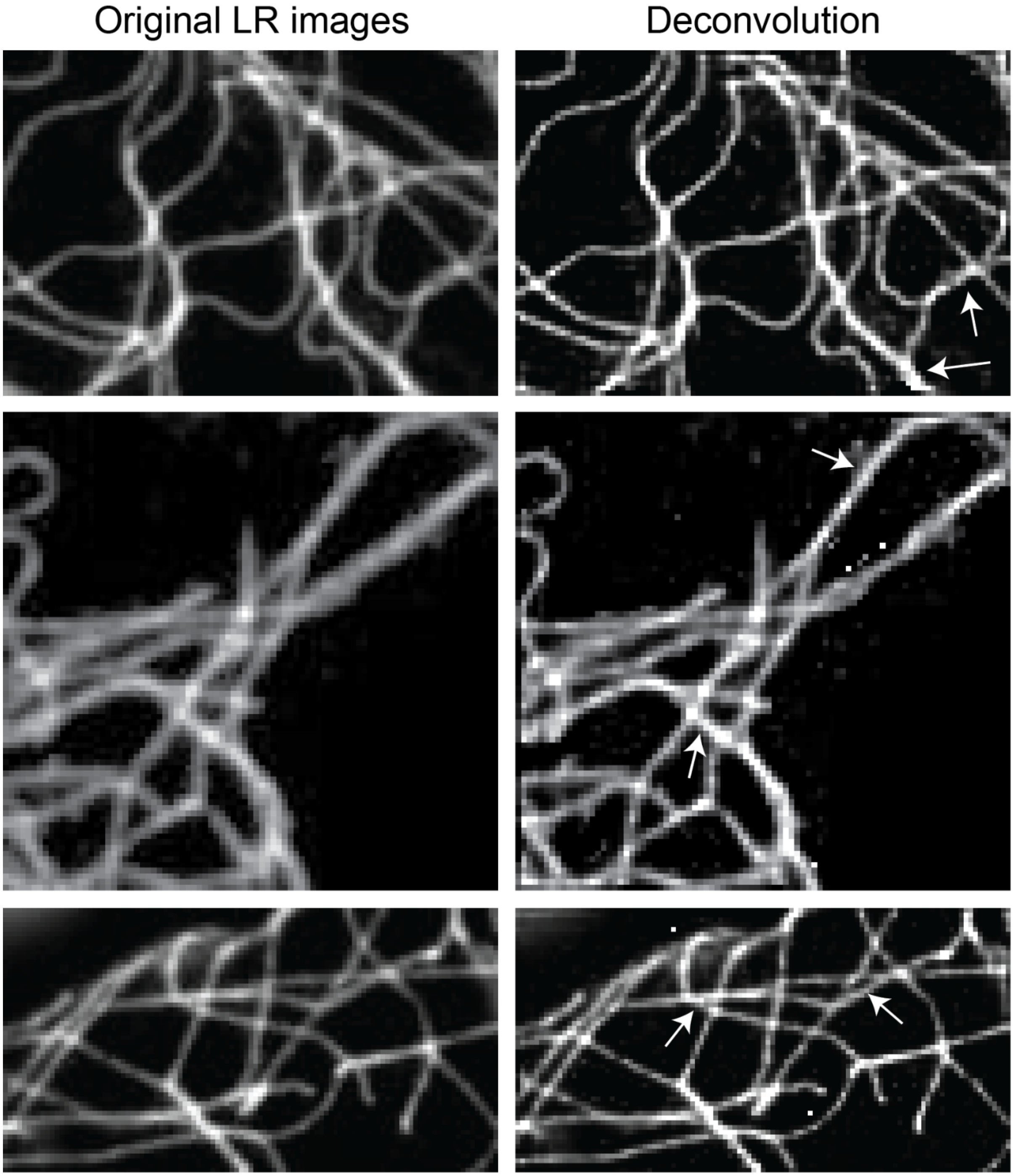

Supplementary Figure 6. Comparison with image deconvolution. Left column, from top to bottom, original LR images as in Figure 3a, e, j in the main text, respectively. Right column, these images after deconvolution, respectively. Comparing the regions indicated by the arrows with the super-resolved image in Figure 3i-vi, we can see that although deconvolution is able to sharpen the image, it cannot resolve overlapping structures, which remain indiscernible (An exception is noted in region indicated by the rightmost arrow in the bottom right image, but in that case the structure is already recognizable in the LR image before deconvolution). The deconvolution is done using a lab-written MATLAB script based on the Richardson-Lucy deconvolution algorithm.

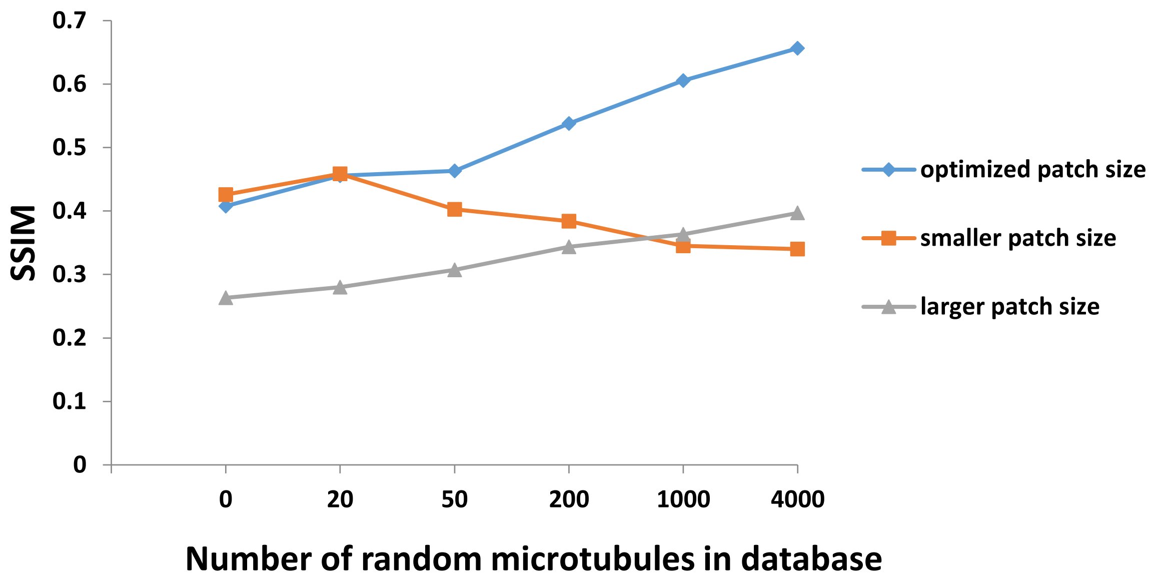

Supplementary Figure 7. Performance of the method with varying patch sizes. Structural similarity index measurement (SSIM) as a function of the number of random microtubules in the database (from 0 to 4000) with different patch sizes at (as used in this paper: LR, 21 pixels X 21 pixels, SR, 15 pixels X 15 pixels, blue curve), (smaller patch size: LR, 5 pixels X 5 pixels, SR, 3 pixels X 3 pixels, orange curve), and (larger patch size: LR, 29 pixels X 29 pixels, SR, 23 pixels X 23 pixels, orange curve), respectively.
